# Unmarried Sri Lankan youth: sexual behaviour and contraceptive use

**DOI:** 10.1186/s40834-022-00185-w

**Published:** 2022-09-14

**Authors:** Malith Kumarasinghe, W. Indralal De Silva, Ranjith de Silva, M. Suchira Suranga

**Affiliations:** 1 Epidemiology Unit, Ministry of Health, No 54/A, New Jayaweera Road, Ethul-Kotte, Sri Jayawardhanapura Kotte, Colombo, 10100 Sri Lanka; 2 University of Colombo, Colombo, Sri Lanka; 3 Independant Investigator, Colombo, Sri Lanka; 4 Family Planning Association, Colombo, Sri Lanka

**Keywords:** Sexual Behaviour, Contraception, Unmarried, Youth, Sri Lanka

## Abstract

**Background:**

Youth are at high risk for casual and unprotected sexual activities even before marriage. The objective of the study is to describe the sexual behavior, and contraceptive use among unmarried youth of Sri Lanka and to assess the factors associated with sexual behaviour.

**Methods:**

An observational descriptive cross-sectional study was conducted in three selected districts in Sri Lanka from 1st March 2019 to 31st January 2020 among 1057 never-married youth using a self-administered questionnaire. Both stratified cluster sampling and snowball sampling were used to select the eligible never-married youth. Factors associated with sexual intercourse were assessed using logistic regression.

**Results:**

Compared to boys (26%), more girls (35%) were engaged in unprotected sexual intercourse. Among sexually active unmarried youth aged less than 20 years, 10% had sexual intercourse with an unknown person. Unmarried Tamil and estate sector youth displayed significantly lower chances of sexual intercourse compared to Sinhala and urban counterparts (OR = 0.390, CI = 0.213-0.715, *p* = 0.002 and OR = 0.807, CI = 0.709-0.978, *p* = 0.020 respectively). Youth in the rural (69.5%) and urban sectors (87.3%) tend to use contraceptives during intercourse compared to the youth in the Estate sector (51.1%).

**Conclusions:**

A significant portion of youth are exposed to sexual risk behavior including unprotected sexual intercourse even before marriage which can contribute to many social and health consequences. Focus interventions are needed to address the issue.

**Supplementary Information:**

The online version contains supplementary material available at 10.1186/s40834-022-00185-w.

## Plain Language Summary

With early puberty and late marriage, Sri Lankan youth are more susceptible to unprotected and casual sex. The physical, psychological, and sociocultural impact of resulting pregnancies and Sexually Transmitted Diseases including HIV could be detrimental. In Sri Lanka, significant variation in sexual intercourse and concurrent use of contraceptive use was observed among different ethnic groups and sectors. Increased popularity for emergency contraceptive pills was observed among never-married female youth. Younger youth (teenagers) tend to engage in more unprotected sexual intercourse compared to older youth in Sri Lanka. Concentrating on reducing barriers to contractive access for younger youth in Sri Lanka is important. To prevent unwanted pregnancies, and Sexually Transmitted Diseases including HIV, targeted interventions are required especially for Tamil ethnic groups and estate sector youth populations in Sri Lanka.

**Supplementary Information:**

The online version contains supplementary material available at 10.1186/s40834-022-00185-w.

## Introduction

Sex is exclusively a biological function, defined based on that individual’s individuality and socio-cultural background. It is biologically associated with male and female reproductive organs. Sex is a complex process to study because it touches upon so many different complex aspects related to human experience. There are four major characteristics of sex frequently focused on by many researchers, namely sexual acts, sexual partners, sexual meanings, and sexual desire and pleasure [1]. Though all four of these elements are important, the first two deserve more attention. As these two elements are the most personal and include activities that may occur in an individual’s life. Sexual desire and pleasure are more difficult aspects to capture which makes the study of that aspect of sex rather problematic. Human behaviour is difficult to comprehend and drawing inferences about the study of sexual behaviour is further complicated by issues such as privacy, acceptability, and appropriateness [2].

Contraception can be defined as the deliberate use of artificial methods or other techniques to prevent pregnancy as a consequence of sexual intercourse. The major forms of artificial contraception are natural methods such as withdrawal or standard days method; barrier methods, of which the commonest is the condom or sheath; the contraceptive pill, contains synthetic sex hormones which prevent ovulation in the female; long-acting and reversible methods such as implants and intrauterine devices, which prevent the fertilized ovum from implanting in the uterus; and male or female sterilization [3]. The use of contraceptive methods in the context of planning a family is referred to as a key component in family planning. The use of contraceptive methods by unmarried women or girls is not normally considered as family planning. Family planning allows people to attain their desired number of children and determine the spacing of pregnancies. It is achieved using contraceptive methods and the treatment of infertility [4].

**Fig. 1:**
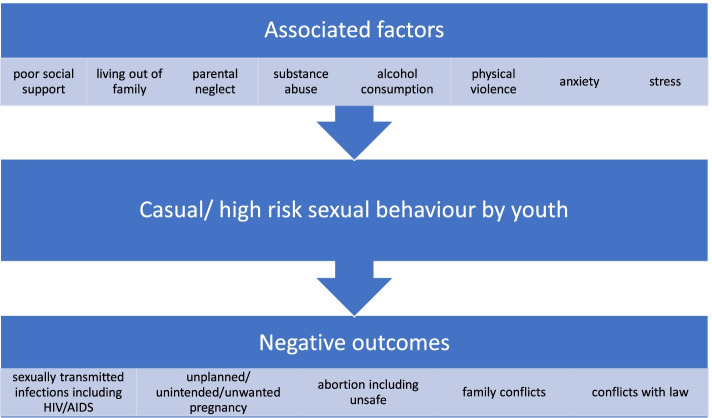
Factors and negative outcomes associated with casual/ high risk sexual behaviour of youth

Globally, there are 1.8 billion adolescents and youth, composing 25% of the world’s population [5]. While many adolescents and youth choose to delay sexual initiation, a significant number are sexually active and desire to prevent or delay pregnancy for multiple years—until completion of education, gaining employment, getting married, or to space their children. Global efforts to prevent unintended & unwanted pregnancies and improve pregnancy spacing among adolescents and youth will reduce maternal and infant morbidity and mortality, decrease rates of unsafe abortion, decrease HIV/STI incidence, improve nutritional status, keep girls in school, improve economic opportunities, and contribute toward reaching the Sustainable Development Goals. Similarly, Paragraph 7.44 of the ICPD Programme of Action urged governments to address adolescent sexual and reproductive health issues, including unwanted pregnancy, unsafe abortion, and sexually transmitted infections including HIV/ AIDS. It also called for the reduction in adolescent pregnancies [6]. Poor social support, living out of family, parental neglect, substance abuse, and alcohol consumption, physical violence, anxiety and stress were identified to be associated with high risk or casual sexual behaviour among adolescents and youth [7–9] (Fig. 1).

In Sri Lanka almost 5% of the pregnancies in 2016 were reported from teenage mothers (less than 20 years). However, the number and percentage of teenage pregnancies reported in the country show a declining trend from 6% in 2012 to 3.9% in 2021. The Annual Report by Family Health Bureau in 2019 revealed that the sub-national disparities of teenage pregnancies are higher in Sri Lanka ranging from 8.9% in Trincomalee to 3.2% in Colombo [10]. The national family planning programme review conducted in 2016 pointed out that a considerable proportion of unmarried young persons in Sri Lanka are sexually active. The National Youth Health Survey 2012/2013 found that around 15% of the respondents declared they had sexual intercourse during the preceding year, of them 5.3% were unmarried [11, 12]. A Needs Assessment Survey on Sexual and Reproductive Health for youth in the Technical and Vocational Education and Training Sector in Sri Lanka in 2015 showed that one-third of the respondents aged 15-29 had engaged in sexual intercourse [12]. This is further complicated by irregularities within the legal system of Sri Lanka as reported in a newspaper indicated that 50 registrars had been interdicted for falsifying marriage registers and solemnizing underage marriages [13].

Following the National youth health survey conducted in 2012-2013, a large-scale or national level information is lacking on sexual behaviour and contraceptive youth of Sri Lanka [11]. Among the few smaller studies conducted subsequently on sexual behaviour of youth, have highlighted the rise in high-risk sexual behaviour among the youth [14, 15]. Further, authorities have observed a recent rise of HIV/AIDS incidence among youth in Sri Lanka [16]. These factors necessities the need to conduct a large population level study on sexual behaviour and contractive of Sri Lankan youth.

The aim of this investigation is the describe the sexual behavior, contraceptive use among unmarried youth of Sri Lanka and to assess the factors associated with sexual behaviour and contraceptive use.

## Methods

Observational descriptive crosse-sectional study was conducted in three selected districts in Sri Lanka from 1st of March 2019 to 31st of January 2020.

### Sampling technique

Three districts were conveniently selected to represent the rural (Hambantota), estate (Nuwara-Eliya) and urban (Puttalam) sectors out of 25 districts in Sri Lanka as the study setting. Only unmarried youth representing the specific sector were recruited in each of the selected district (e.g. only estate sector youth from Nuwara-eliya, urban sector youth from Puttalam etc.) after receiving oral informed consent. Mixed sampling method was used to select never-married youth from these districts due to complexity of the study problem and difficulty in capturing representative sample from the study population. Both probability and non- probability sampling techniques were used together. Stratified cluster sampling (probability sampling) with snow-ball sampling (non-probability sampling) was used to select the eligible never married youth (Additional files 1 and 2: Annexure 1 and 2). We opted to capture the never married youth outside their place of residence due to socio-cultural factors and difficulty in conducting household survey. Youth away from their respective households, respond more genuinely, to report their intimate relationships, particularly their experience in love affairs and related information, if appropriate survey techniques were used. Therefore, to collect reliable information from the target group of youth in Sri Lanka, youth were selected from various vocational and training centers, higher educational institutions and registered tuition classes using stratified cluster sampling and through NGOs using snowball technique, of the selected districts. In addition, the research team decided to adopt the self-completion type of questionnaire administration, as the suitable survey technique. The questionnaire was administered, consequent to it being introduced to the group. The eligible respondents were requested to arrive at particular centers for the self-reporting of the above-mentioned survey questionnaire. We used self-administered survey method as the respondents tend to provide positive and culturally desirable answers in interviewer administered technique than in self-administered. Therefore, socially undesirable behaviours are likely to be under reported in interviewer administered surveys compared to self-administered ones [17].

It was decided to recruit a sample of 1100 never married youth of age 15-24 years as the total sample size from 3 districts of the study. By Stratified Cluster sampling 780 were planned to be recruited (Additional files 3 and 4: Annexure 3 and 4) and by snowball technique, investigation team decided to include 320 participants.

#### Stratified cluster sampling

It was decided to include 260 never married youth from each district through probability proportionate to size (PPS) sampling using stratified cluster sampling as cluster size was 20. Thus, total of 780 never-married youth was selected (Additional files 3 and 4: Annexure 3 and 4).

#### Snowball technique

Sample of 320 was recruited by snowball technique in these 3 districts. Allocation of the target samples for each district was based on the actual proportion of never married youth population of age 15-24 years observed in 2012 population census. It has been assumed that those proportions have not changed by 2019 significantly. Thus, from Hambantota it was decided to recruit 64, from Nuwara-Eliya-101 and from Puttalam-155. Non-Governmental Institutions who were working with youth in these districts were used to recruit the eligible youth. These NGOs were used as the place of completion of the questionnaire (Additional file 4: Annexure 4).

Number of respondents who were successfully recruited and completed the questionnaire were 305 from rural district, 345 from estate district and 407 from urban district (*N* = 1057) (Additional file 4: Annexure 4).

Data were coded and analysed using Statistical Package for Social Sciences (SPSS) 22 version. Rates were presented as proportions. Factors associated with the sexual intercourse was assessed using unadjusted and adjusted Odds Ratios. All variables were used to perform the multivariate analysis using logistic regression (enter method) irrespective of their significance in bivariate analysis to identify adjusted ORs and their Confidence Intervals [18].

Ethical clearance was obtained from Ethical Review Committee of ChildFund International.

### Respondent and public involvement statement

Research team actively discussed with the unmarried youth during the decision-making process on selection between interviewer administered or self-administered questionnaire. Unmarried youth preferred self-administered questionnaire informing the team that they are comfortable with it as reduces the fear of judgment by the interviewer and increases the ability to provide responses. Further, the respondents were in favour of selecting the place of data collection outside their residence. They felt discomfort answering sensitive questions at their place of residence in the presence of close family members despite the assurance of selection of place of privacy in their households. This led to the complex sampling techniques as mentioned in the methods section. The summary of findings was made available at places of data collection, namely vocational training centers, universities, workplaces, non-governmental organizations for the use of both respondents and administrators of these places.

## Results

Out of 1100 unmarried youth of age 15 -24 years participated in providing required information for the self-completion type of questionnaire that was administered, 1057 (96%) respondents completed the questionnaire to acceptable level, and they were taken for detail analysis in subsequent sections (Additional file 4: Annexure 4).

### Characteristics of youth respondents

Approximately equal age-wise representation was observed whereas slightly higher female representation was reported (44.7% vs. 55.3%). Majority (91.2%) of the youth were residing with their parents in consistent with the sociocultural norms of Sri Lanka. Most of the respondents (58.8%) belonged to Sinhala ethnicity which is the major ethnic group in Sri Lanka. Only 10% of the youth were employed whereas close to 20% of the youth were searching for employment (Additional file 5: Annexure 5).

Majority (56.5%) of the respondents were aware of peers having sexual intercourse. Close to two third of the respondents (64.1%) who had experience in sexual intercourse were boys. Compared to male youth, more females were engaged in unprotected sexual intercourse among the study respondents (31/118 vs. 24/68) (Additional file 6: Annexure 6).

Irrespective of age or sex, the majority of the youth engage in sexual intercourse with their lovers. Around 10% of teenagers (age 15-19) had sexual intercourse with an unknown person. In addition, around 10% of the male youth were engaged in sexual intercourse with a commercial sex worker whereas it was less than 2% among female respondents (Additional file 7: Annexure 7).

Significant association was found with gender and sexual intercourse among unmarried youth. More unmarried boy (25%)s were found to be engaged in premarital sexual intercourse compared to girls (11%) in our study population (*p* <  0.01). In addition, age of the respondent as well as the age of the respondent’s mother were significantly associated with ever engaged in sexual intercourse among unmarried youth (Table 1).

**Table 1:** Association of Demographic characteristics with ever engaged in sexual intercourse among unmarried youth

Variable	Ever engaged in sexual intercourse						Test statistics	Sig.
	Yes		No		Total			
	No	%	No	%	No	%		
Age group								
20-24 years	100	18.4	442	81.6	542	100.0	χ^2^ = 0.84	0.359
15-19 years	84	16.3	431	83.7	515	100.0		
Age in years	19.95 (2.34)		19.52 (2.33)		19.59 (2.33)		t = 2.26	0.024*
Sex								
Female	66	11.3	519	88.7	585	100.0	χ^2^ = 34.19	< 0.01**
Male	118	25.0	354	75.0	472	100.0		
Mother’s age								
< 40 years	31	16.8	154	83.2	185	100.0	χ^2^ = 7.46	0.059
40-49 years	80	14.9	457	85.1	537	100.0		
50-59 years	64	21.3	237	78.7	301	100.0		
60+ years	9	26.5	25	73.5	34	100.0		
Mother’s age	47.21 (7.42)		45.80 (6.74)		46.05 (6.88)		t = 2.52	0.012*
Having siblings								
None	4	8.3	44	91.7	48	100.0	χ^2^ = 2.88	0.090
One or more	180	17.8	829	82.2	1009	100.0		
Total	184	17.4	873	82.6	1057	100.0		

*Continuous variables: mean (SD)*

*p <  0.05, **p <  0.01

Ethnicity and time spent on communication devices per day was associated with the engagement of sexual intercourse among unmarried youth. Moor (24.5%) and Sinhalese (19.6%) youth demonstrated higher engagement in sexual intercourse than Tamil ethnicity (9.4%, *p* <  0.01). Similarly increase in time spent on communication devices increase the chances of pre-marital/ casual sexual intercourse. Significantly higher proportion (23.5%) of youth who spend spend more than 2 hours per day with communication devices tend to report a history of sexual intercourse compared to the youth who spent less than half an hour per day on communication devices (11.3%*p* = 0.007). Further, identical pattern was demonstrated on sexual intercourse by unmarried youth on time spent on online content with spending more than 2 hours per day showing 23.9% engagement of sexual intercourse compared to 12.4% who spent less than 30 minutes per day (*p* = 0.019) (Table 2).

**Table 2:** Association of Socio-economic characteristics with ever engaged in sexual intercourse among unmarried youth

Variable	Ever engaged in sexual intercourse						Test statistics	Sig.
	Yes		No		Total			
	No	%	No	%	No	%		
Ethnicity								
Sinhala	122	19.6	499	80.4	621	100.0	χ^2^ = 20.13	< 0.01**
Tamil	28	9.4	269	90.6	297	100.0		
Moor	34	24.5	105	75.5	139	100.0		
Sector								
Urban	82	20.1	325	79.9	407	100.0	χ^2^ = 5.65	0.059
Rural	55	18.0	250	82.0	305	100.0		
Estate	47	13.6	298	86.4	345	100.0		
Level of education								
Grade 1-10	9	16.7	45	83.3	54	100.0	χ^2^ = 2.96	0.398
Passed GCE(O/L)	83	18.2	374	81.8	457	100.0		
Passed GCE(A/L)	47	14.6	274	85.4	321	100.0		
Technical/Dip/Undergraduate/Degree	45	20.0	180	80.0	225	100.0		
Family environment								
Positive	147	17.7	683	82.3	830	100.0	χ^2^ = 0.25	0.619
Negative	37	16.3	190	83.7	227	100.0		
Time spent on online content per day								
< 30 minutes	27	12.4	190	87.6	217	100.0	χ^2^ = 12.10	0.007**
30-59 minutes	63	15.4	345	84.6	408	100.0		
60-119 minutes	39	19.3	163	80.7	202	100.0		
120+ minutes	55	23.9	175	76.1	230	100.0		
Time spent on online content per day (minutes)	91.68 (99.65)		71.56 (106.32)		75.06 (105.42)		t = 2.36	0.019*
Time spent to communicate per day								
< 30 minutes	36	11.3	283	88.7	319	100.0	χ^2^ = 15.71	0.001**
30-59 minutes	52	19.3	217	80.7	269	100.0		
60-119 minutes	36	16.8	178	83.2	214	100.0		
120+ minutes	60	23.5	195	76.5	255	100.0		
Time spent to communicate per day (minutes)	119.03 (161.09)		77.42 (118.92)		84.66 (128.16)		t = 4.03	0.019*
Current economic activity								
Employed	29	23.2	96	76.8	125	100.0	χ^2^ = 3.71	< 0.001**
Student	113	17.1	548	82.9	661	100.0		
Searching a job	30	15.2	168	84.8	198	100.0		
Idling	12	16.4	61	83.6	73	100.0		
Total	184	17.4	873	82.6	1057	100.0		

*Continuous variables: mean (SD)*

*p < 0.05, **p < 0.01

Use of any substance including either alcohol or illicit drugs was associated with engagement of sexual intercourse among unmarried youth respondents with more than 20 percentage point rise in sexual intercourse among youth substance users (32.0% vs. 10.6%, *p* <  0.001). Level of knowledge and ever engaging in a love affair were linked with having sexual intercourse among unmarried youth. (*p* <  0.001 and 0.007 respectively) (Table 3).

**Table 3:** Association of sexual and reproductive health knowledge & behavior with ever engaged in sexual intercourse among unmarried youth

Variable	Ever engaged in sexual intercourse						Test statistics	Sig.
	Yes		No		Total			
	No	%	No	%	No	%		
Use of any substance								
Yes	108	32.0	229	68.0	337	100.0	χ^2^ = 73.45	< 0.001**
No	76	10.6	644	89.4	720	100.0		
Ever had love affair								
Yes	174	18.5	766	82.5	940	100.0	χ^2^ = 7.18	0.007**
No	10	8.5	107	91.5	117	100.0		
Overall knowledge on SRH								
Low	12	5.7	198	94.3	210	100.0	χ^2^ = 47.50	< 0.001**
Medium	86	15.7	461	84.3	547	100.0		
High	86	28.7	214	71.3	300	100.0		
Overall knowledge on SRH (out of 100)	63.64 (18.47)		50.35 (19.72)		52.66 (20.14)		t = 8.40	< 0.001**
Aware of sexual intercourse among peers								
Yes	143	32.1	303	67.9	446	100.0	χ^2^ = 115.26	< 0.001**
No	41	6.7	570	93.3	611	57.8		
Total	184	17.4	873	82.6	1057	100.0		

Continuous variables: mean (SD), **p < 0.01

Results shows that the Tamils respondents tend to report 61% lower chance of having a history of sexual intercourse compared to the Sinhalese (OR = 0.390, CI = 0.213-0.715, *p* = 0.002). Similarly, the respondents who are residents in the estate sector show a 20% lower chance of having premarital sexual intercourse compared to their urban counterparts (OR = 0.807, CI = 0.709-0.978, *p* = 0.020). Similarly, significant reduction in premarital/ causal sexual intercourse was observed among the youth who do not use substances such as alcohol and illicit drugs (OR = 0.419, CI = 0.267-0.658, *p* <  0.001). Zero awareness of sexual intercourse among peers points toward a significant reduction in engaging in sexual intercourse among unmarried youth (OR = 0.224, CI = 0.149-0.337, *p* <  0.001) (Table 4).

**Table 4:** Factors associated with ever engaging in sexual intercourse among unmarried youth

Variable	Level	Ever engaged in sexual intercourse				Univariate Odd Ratio (95% CI, Probability)	Multivariate Odd Ratio (95% CI, Probability)
		Yes		No			
		No	%	No	%		
Age in group	20-24 years	100	18.5	442	81.5		
	15-19 years	84	16.3	431	83.7	0.861 (0.626-1.185, *p* = 0.359)	0.903 (0.547-1.493, *p* = 0.691)
Sex	Female	66	11.3	519	88.7		
	Male	118	25.0	354	75.0	2.621 (1.884-3.647, *p* < 0.001)	1.378 (0.857-2.214, *p* = 0.186)
Mother’s age	< 40 years	31	16.8	154	83.2		
	40-49 years	80	14.9	457	85.1	0.870 (0.553-1.368, *p* = 0.546)	0.774 (0.451-1.329, *p* = 0.353)
	50-59 years	64	21.3	237	78.7	1.341 (0.835-2.156, *p* = 0.225)	1.098 (0.596-2.023, *p* = 0.764)
	60+ years	9	26.5	25	73.5	1.788 (0.761-4.201, *p* = 0.182)	1.929 (0.642-5.792, *p* = 0.242)
Having siblings	None	4	8.3	44	91.7		
	One or more	180	17.8	829	82.2	2.388 (0.847-6.732, *p* = 0.100)	2.820 (0.902-8.811, *p* = 0.075)
Ethnicity	Sinhala	122	19.6	499	80.4		
	Tamil	28	9.4	269	90.6	0.426 (0.275-0.659, *p* < 0.001)	0.390 (0.213-0.715, *p* = 0.002)
	Moor	34	24.5	105	75.5	1.324 (0.858-2.045, *p* = 0.205)	1.329 (0.784-2.252, *p* = 0.291)
Sector	Urban	82	20.1	325	79.9		
	Rural	55	18.0	250	82.0	0.872 (0.597-1.274, *p* = 0.479)	1.279 (0.810-2.020, *p* = 0.290)
	Estate	47	13.6	298	86.4	0.625 (0.423-0.925, *p* = 0.019)	0.807 (0.709-0.978, *p* = 0.020)
Level of education	Grade 1-10	9	16.7	45	83.3		
	Passed GCE(O/L)	83	18.2	374	81.8	1.110 (0.522-2.359, *p* = 0.787)	0.962 (0.394-2.345, *p* = 0.931)
	Passed GCE(A/L)	47	14.6	274	85.4	0.858 (0.393-1.870, *p* = 0.700)	0.576 (0.219-1.516, *p* = 0.264)
	Technical/Dip/Undergraduate/Degree	45	20.0	180	80.0	1.250 (0.569-2.745, *p* = 0.578)	0.848 (0.315-2.279, *p* = 0.743)
Family environment	Positive	147	17.7	683	82.3		
	Negative	37	16.3	190	83.7	0.905 (0.610-1.343, *p* = 0.619)	0.648 (0.405-1.036, *p* = 0.070)
Time spent on online content per day	< 30 minutes	27	12.4	190	87.6		
	30-59 minutes	63	15.4	345	84.6	1.285 (0.792-2.086, *p* = 0.310)	1.350 (0.754-2.418, *p* = 0.312)
	60-119 minutes	39	19.3	163	80.7	1.684 (0.988-2.870, *p* = 0.056)	1.380 (0.735-2.589, *p* = 0.316)
	120+ minutes	55	23.9	175	76.1	2.212 (1.336-3.662, *p* = 0.002)	1.467 (0.794-2.710, *p* = 0.221)
Time spent to communicate per day	< 30 minutes	36	11.3	283	88.7		
	30-59 minutes	52	19.3	217	80.7	1.884 (1.189-2.985, *p* = 0.007)	1.415 (0.828-2.419, *p* = 0.204)
	60-119 minutes	36	16.8	178	83.2	1.590 (0.966-2.618, *p* = 0.068)	1.110 (0.622-1.981, *p* = 0.724)
	120+ minutes	60	23.5	195	76.5	2.419 (1.540-3.800, *p* < 0.001)	1.632 (0.953-2.793, *p* = 0.074)
Current economic activity	Employed	29	23.2	96	76.8		
	Student	113	17.1	548	82.9	0.683 (0.430-1.084, *p* = 0.105)	0.715 (0.403-1.267, *p* = 0.251)
	Searching for a job	30	15.2	168	84.8	0.591 (0.335-1.044, *p* = 0.070)	0.633 (0.322-1.245, *p* = 0.185)
	Idling	12	16.4	61	83.6	0.651 (0.309-1.372, *p* = 0.259)	0.617 (0.248-1.536, *p* = 0.300)
Use of any substance	Yes	108	32.0	229	68.0		
	No	76	10.6	644	89.4	0.250 (0.180-0.348, *p* < 0.001)	0.419 (0.267-0.658, *p* < 0.001)
Ever had love affair	Yes	174	18.5	766	81.5		
	No	10	8.5	107	91.5	0.411 (0.211-0.803, *p* = 0.009)	0.449 (0.211-0.954, *p* = 0.037)
Overall knowledge on SRH	Low	12	5.7	198	94.3		
	Medium	86	15.7	461	84.3	3.078 (1.645-5.759, *p* < 0.001)	2.493 (1.256-4.947, *p* = 0.009)
	High	86	28.7	214	71.3	6.631 (3.517-12.502, *p* < 0.001)	4.284 (2.130-8.618, *p* < 0.001)
Aware of sexual intercourse among peers	Yes	143	32.1	303	67.9		
	No	41	6.7	570	93.3	0.152 (0.105-0.222, *p* < 0.001)	0.224 (0.149-0.337, *p* < 0.001)

Smaller proportion of youth aged less than 20 years used contraception during premarital or casual sexual intercourse compared to older youth (59.5% vs 79.0%). Estate sector youth reported lower percentages of use of contraceptives during intercourse compared to both urban and rural sector unmarried youth (51.1% vs 69.5% & 87.3% respectively). (Additional file 8: Annexure 8)**.** The most common method of contraception among unmarried youth in Sri Lanka, irrespective of age is condoms. The overall use of Postinor (emergency contraceptive pills) is close to one in five unmarried female youth (19.7%) (Additional file 9: Annexure 9).

## Discussion

This study demonstrated a significantly lower probability of never-married Tamil and estate sector youth experiencing sexual intercourse compared to Sinhalese and urban counterparts (OR = 0.390, CI = 0.213-0.715, *p* = 0.002 and OR = 0.807, CI = 0.709-0.978, *p* = 0.020 respectively). There could be many factors contributing to the possibility of lower engagement in sexual intercourse among unmarried Tamil youth. One element could be the living condition. Most of our Tamil respondents belonged to the estate sector (> 90%). Therefore, these youth are living in a very restrictive society with limited out of neighbourhood interactions. They are usually either educated at estate school or employed at estates. This could have limited the chances of experiencing new relationships and thereby sexual interactions than Sinhala youth [19, 20].

Present study findings describe a lower probability of premarital sexual intercourse among the youth who do not engage in substance abuse or alcohol consumption compared to youth who use. Higher chances of sexual activities including casual and unprotected were reported by several studies globally among the youth with substance abuse. Findings of this study point toward reinforcing this fact [21–24]. Similarly, the present study revealed that awareness of sexual intercourse among peers significantly increases the probability of engaging in sexual intercourse although the causality is not clear as this is contrary to the majority of existing literature globally [9, 25]. However, few studies have concluded that the close environment and neighbourhood which induce or promote premarital or casual sexual relationships, could encourage the youth within the said neighbourhood to practice the same [26–28].

A smaller proportion of youth aged less than 20 years used contraception during premarital or casual sexual intercourse compared to older youth (59.5% vs 79.0%). Does this point towards existing barriers in access to contraception for underaged youth in Sri Lanka? Sri Lanka being a conservative country, engaging in premarital or casual sexual activity is discouraged by the community including the religious leaders. Premarital sexual intercourse among school-going youth could result in the expulsion of the youth from the respective school in Sri Lanka. Though a concentrated effort was made during the past decade to reduce such occurrences, it is not an uncommon scenario especially for the female youth to seek a different school following the negative publicity. Similarly, it could be extremely difficult for an unmarried youth aged less than 18 years to obtain contraceptives from pharmacies and groceries due to the fear of rejection and being condemned by the community including the seller. As Suranga, De Silva and Kumarasinghe reported in 2020, some pharmacists request proof from youth to confirm their age before selling contraceptives in Sri Lanka, although it is not a regulatory requirement [29]. According to the present study, the bottlenecks for access to contraception seem to be worse in the estate sector as estate sector youth reported lower percentages of use of contraceptives during intercourse compared to both urban and rural sector never-married youth. Issues pertaining to access to contraceptives in the estate sector could have contributed to the low usage of contraceptives as most of the pharmacies and supermarkets are concentrated in urban areas which require long traveling time for youth in the estate sector [30].

Emergency Contraceptives were introduced in Sri Lanka in 2008 as a drug that can be purchased from a registered pharmacy without having a prescription from a medical practitioner. Since the time of introduction, the use of emergency contraception increased among Sri Lankan women every year. It can be assumed that over 3 million Emergency Contraceptive Pills are used by women in Sri Lanka annually [31]. The overall use of Postinor which is a trade name for commonly used emergency contraceptive pills in Sri Lanka is comparatively high among never married female youth. On average, close to one in five unmarried female youth have used Postinor following sexual intercourse as the preferred method of contraception is a cause of concern [31]. This high use points toward the high-risk nature of sexual intercourse among these females. Therefore, further exploration of the causes for this high usage needs to be conducted. Further studies could be conducted to identify the pattern of emergency contractive usage and whether they are being used as an alternative to regular contraceptive methods. Exposure to high doses of hormones, in long term, could be harmful [32].

We conducted a robustness check of the binary logistic regression (enter method) in the statistical analysis of associated factors using a generalized linear model (binary probit). The results were similar except for two variables namely “whether or not having siblings” and “ever had love affairs” (*p* = 0.075 vs 0.045, *p* = 0.037 vs 0.055; binary logistic regression vs generalized linear model respectively).

## Limitations

Use of unconventional mixed method for sampling due to difficulty in conducting the study in either schools or in households and to increase the representativeness of the study sample could have introduced bias. Further limiting the study to three districts to represent the urban, rural and estate sector in Sri Lanka could affect the generalizability of the study. This study was conducted immediately prior and early stages of the COVID-19 pandemic. Therefore, the impact of COVID-19 on sexual behaviour and contraceptive use of never-married youth is not reflected in the findings of this study.

## Conclusions

Study concludes that a significant portion of youth expose to sexual risk behavior including unprotected sexual intercourse even before the marriage which can contribute to many social and health consequences. Significant ethnic variability was observed in engagement of sexual intercourse. Sinhalese youth demonstrated higher engagement than Tamils whereas the highest engagement was reported among the Muslims. High volume usage of communication devices and on online content, use of substance and alcohol were identified as risk factors for pre-marital/ casual sexual intercourse among unmarried young persons. Level of knowledge and ever engaging in a love affair were associated with having sexual intercourse among unmarried youth (*p* <  0.001 and 0.007 respectively). Age was negatively associated with the use of contraception during the intercourse. However, use of contraception was low among estate sector never-married youth compared to Sinhalese youth. Preference for emergency contraceptive pills as the method of contraception was observed among female youth. The sexual risk behavior low use of contraception identified in this study shows high demand for focused interventions. National level awareness campaign could be implemented to reduce the use of emergency contraceptives as a routine contraceptive method and to improve the access to modern contraceptives for younger youth. Further studies could be conducted to explore the reasons behind ethnic and sector variability in the engagement of premarital/casual sexual intercourse and the concurrent use of contraceptives.

## Supplementary Information

**Additional file 1: Annexure 1.** Detailed illustration of use of combination of random and convenient sampling techniques for selection of study participants.

**Additional file 2: Fig. 2.** Illustration on how the investigation team ensured adequate representation of never married youth population.

**Additional file 3.** Sample size calculation.

**Additional file 4: Table.** Youth population, sample allocated and respondents by District/ Sector.

**Additional file 5: Table.** Profile of youth respondents of age 15-24.

**Additional file 6: Table.** Sexual behavior and contraceptive use by sex.

**Additional file 7: Table.** Type of partner involved in sexual intercourse among unmarried youth.

**Additional file 8: Table.** Use of contraception and selected variables among unmarried youth among unmarried youth who ever had sexual intercourse (*N* = 184).

**Additional file 9: Table.** Method of contraception used by age and sex among unmarried youth who ever had sexual intercourse.

## Data Availability

Data are available on reasonable request. Raw data without personal identifiers are available from corresponding author on reasonable request due to conditions set by the authorities which granted administrative approval to conduct the study in their respective institutions.
