# Supplementary material for: Unmarried Sri Lankan youth: sexual behaviour and contraceptive use
Source: Contracept Reprod Med. 2022 Sep 14;7:19. doi: 10.1186/s40834-022-00185-w (PMC9471037; doi:10.1186/s40834-022-00185-w)
Supplement: Supplementary file 1 — Additional file 1: Annexure 1. Detailed illustration of use of combination of random and convenient sampling techniques for selection of study participants. [file 40834_2022_185_MOESM1_ESM.pdf]

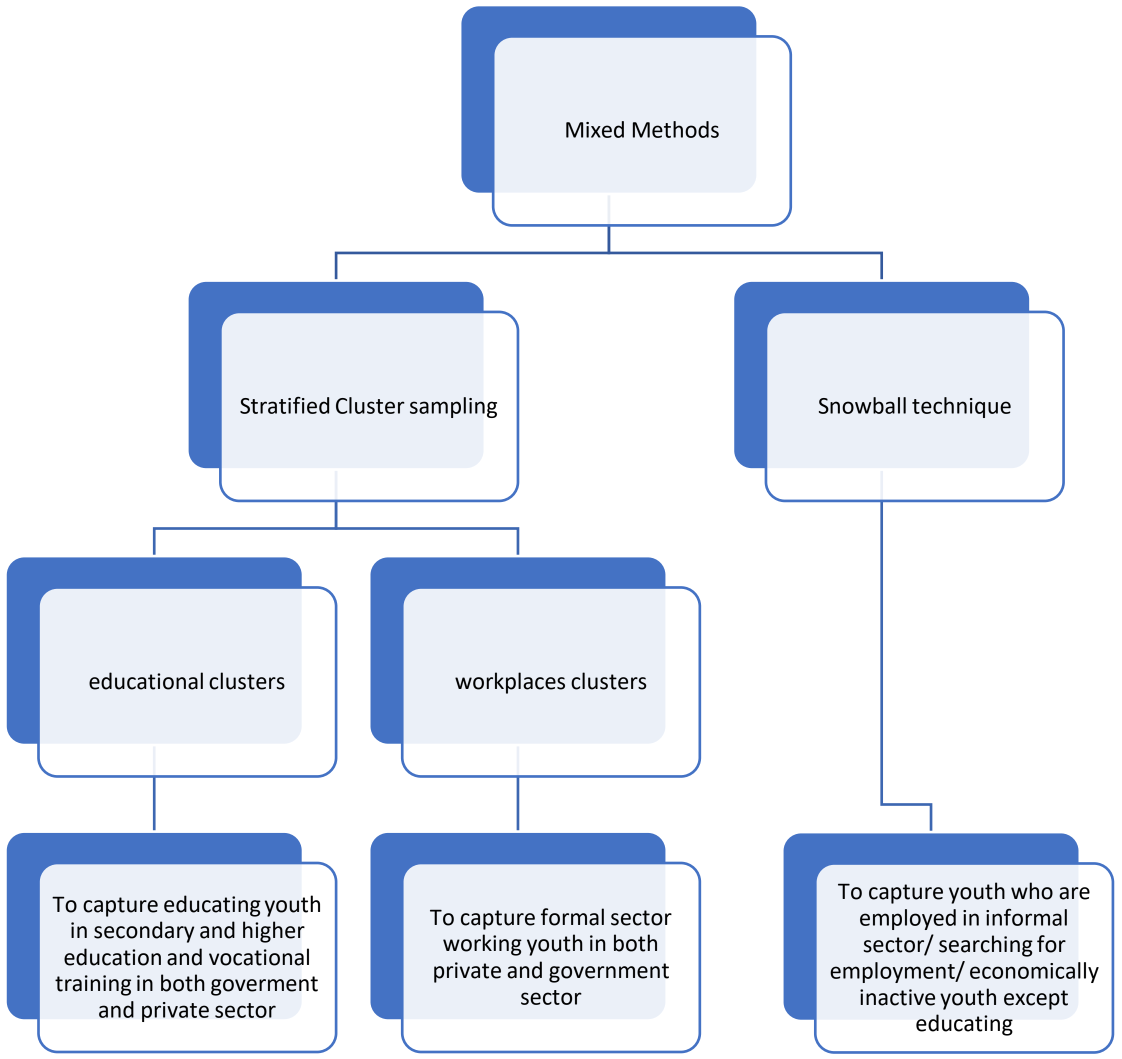

Annexure 1: Detailed illustration of use of combination of random and convenient sampling techniques for selection of study participants
