# Supplementary material for: Unmarried Sri Lankan youth: sexual behaviour and contraceptive use
Source: Contracept Reprod Med. 2022 Sep 14;7:19. doi: 10.1186/s40834-022-00185-w (PMC9471037; doi:10.1186/s40834-022-00185-w)
Supplement: Supplementary file 2 — Additional file 2: Fig. 2. Illustration on how the investigation team ensured adequate representation of never married youth population. [file 40834_2022_185_MOESM2_ESM.pdf]

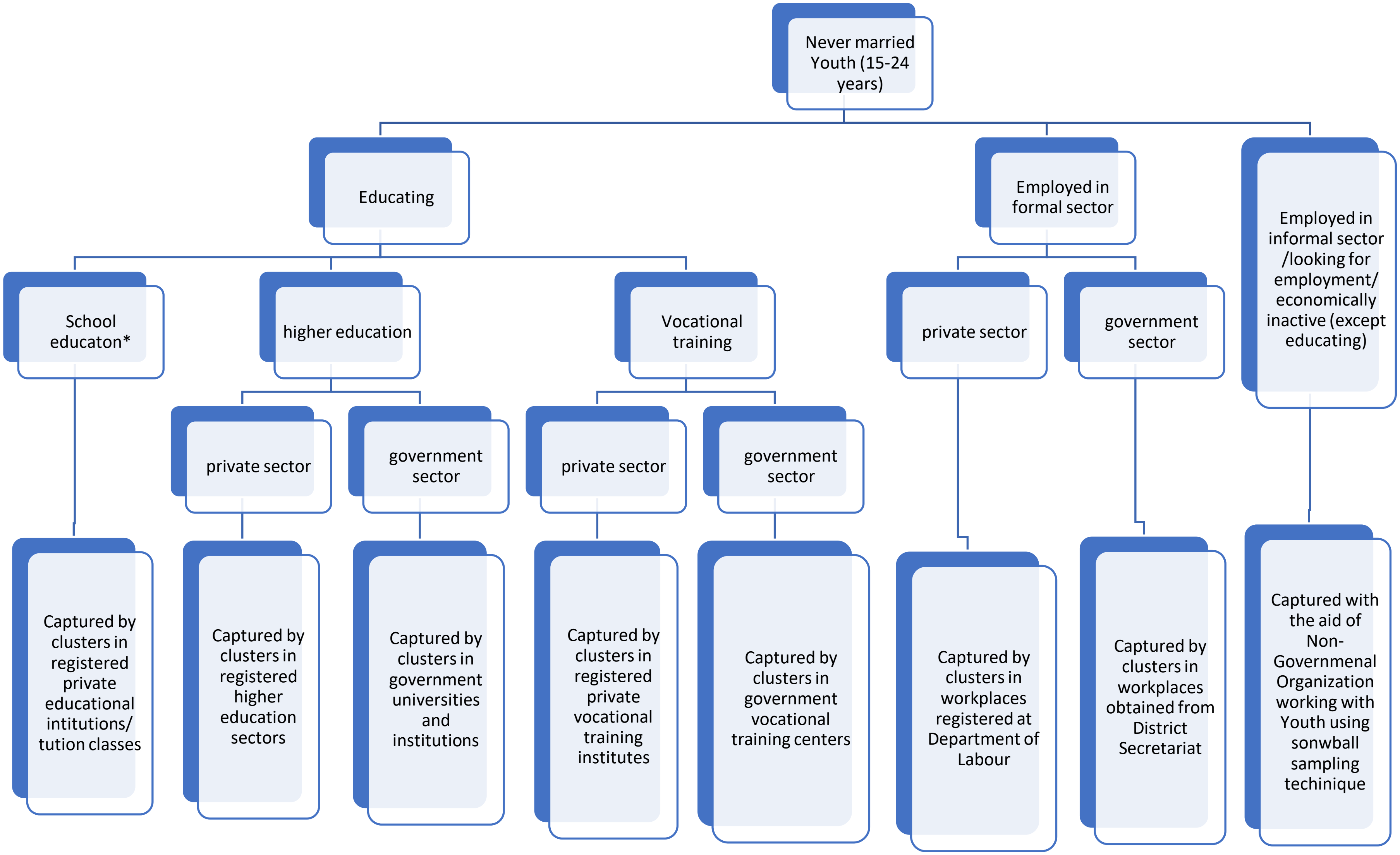

Figure 2: Illustration on how the investigation team ensured adequate representation of never married youth population*

*Due to the sensitive nature of the questions including the questions of sexual activity, resistance from school authorities (which was evident during the implementation of comprehensive SRH education at schools) could have resulted in inability to achieve the required sample size and under representation of such communities in the sample. To overcome this issue, we selected registered private tuition classes and educational intuitions as all most all youth who attend government schools attend theses intuitions for additional knowledge gain.
