# Supplementary material for: Unmarried Sri Lankan youth: sexual behaviour and contraceptive use
Source: Contracept Reprod Med. 2022 Sep 14;7:19. doi: 10.1186/s40834-022-00185-w (PMC9471037; doi:10.1186/s40834-022-00185-w)
Supplement: Supplementary file 3 — Additional file 3. Sample size calculation. [file 40834_2022_185_MOESM3_ESM.docx]

**Sample size calculation**

**Stratified Cluster sampling**

In simple random sampling technique where each unit is drawn randomly from the study universe, the sample size n is given as,

n= (〖z_(α/2)〗^2×P(P-1))/〖(MOE)〗^2 (Lwanga & Lemeshow, 1991)

Where Z = 1.96 (95% confidence level)

P = 0.2 (Proportion of unmarried youth sexually active)

MOE = 0.05 (Margin of error – 5%)

n = Calculated Sample Size

Calculated Sample Size = 245

As cluster sampling method was used in this study, correction to the homogeneity within the cluster was added (Abramson & Abramson, 2008)

N= Design effect x n

Design effect = 1+ δ (β-1)

β= cluster size; taken as 20

δ= rho; in the absence of previous studies, as mentioned by the Bennett and others estimated value of rho was taken as 0.1(Bennett et al., 1991). In a given cluster of a vocational training center, course cohort of a university, private tuition class, private higher educational institutes, youth from families of different socio-economic and cultural backgrounds attend. Hence, there was adequate heterogeneity within clusters that justifies the use of 0.1 as the rho.

Design effect = 1+ 0.1 (20-1) =1.9

Therefore, the required sample size= 384.16 x 1.9 = 710.5

≈ 711 Never married Youth

After adding 5% for non-response,

730 /0.95 = 749

It was decided to include 260 never married youth from each district through probability sampling using stratified cluster sampling as cluster size was 20. Thus, total of 780 never-married youth was selected.

Total No of clusters- 39

Clusters per district- 13

As only 43.2 per cent of 15-24 aged youth were in labour force with majority of economically inactive youth are educating, it was decided to allocate 7 clusters to education institutes and 6 clusters to working places in each district (*Sri Lanka* *Labour Force Survey*, 2019; De Silva, 2020).

Total No of educating youth clusters- 21

Total No of formal sector employed youth clusters- 18

**Definition of a cluster**

Educational Stratum*

Any registered private educational institution (registered at Higher Education Ministry or at Divisional Secretariat) or government institution (excluding schools) with at least 50 registered students physically located within the selected district.

Workplace Stratum*

Any registered private workplace (registered at either Department of Labour or at Divisional Secretariat) or government institution with at least 200 employees physically located within the selected district. (Number of employees as 200 was selected to enable the recruitment of 20 youth in the workplace).

**Due to the sensitive nature of the questions including the questions of sexual activity, resistance from school authorities (which was evident during the implementation of comprehensive SRH education at schools) could have resulted in inability to achieve the required sample size and under representation of such communities in the sample. To overcome this issue, we selected registered private tuition classes and educational intuitions as all most all youth who attend government schools attend theses intuitions for additional knowledge gain.
