# Supplementary material for: Unmarried Sri Lankan youth: sexual behaviour and contraceptive use
Source: Contracept Reprod Med. 2022 Sep 14;7:19. doi: 10.1186/s40834-022-00185-w (PMC9471037; doi:10.1186/s40834-022-00185-w)
Supplement: Supplementary file 4 — Additional file 4: Table. Youth population, sample allocated and respondents by District/ Sector. [file 40834_2022_185_MOESM4_ESM.docx]

**Table: Youth population, sample allocated and respondents by District/ Sector**

| **Total Population/ Sample** | **Hambantota-Rural** | **Nuwara-eliya-Estate** | **Puttalam-Urban** | **Total** |
| --- | --- | --- | --- | --- |
| Total population | 599,903 | 711,644 | 762,396 | 2,073,943 |
| Youth population (15-24 yrs.)  N  % | 93,162  15.5 | 96,950  13.6 | 123,070  16.1 | 313,182  15.1 |
| Never married Youth population  (15-24 yrs.)  N  % | 73,253  78.6 | 81,318  83.9 | 93,503  76 | 248,074  79.2 |
| % distribution of never married youth (15-24 yrs.) | 29.5 | 32.8 | 37.7 | 100.0 |
| **Targeted Total Sample** | **324** | **361** | **415** | **1100** |
| Targeted sample size by probability sampling | 260 | 260 | 260 | 780 |
| In education youth category | 140 | 140 | 140 | 420 |
| In employed youth category | 120 | 120 | 120 | 360 |
| Targeted sample size by nonprobability sampling | 64 | 101 | 155 | 320 |
| **No. of total completed questionnaires** | **305** | **345** | **407** | **1057** |
| Completed questionnaires in probability sampling section | 260 | 260 | 260 | 780 |
| In education youth category | 140 | 140 | 140 | 420 |
| In employed youth category | 120 | 120 | 120 | 360 |
| Completed questionnaires in nonprobability sampling section | 45 | 85 | 147 | 277 |
