# Supplementary material for: Unmarried Sri Lankan youth: sexual behaviour and contraceptive use
Source: Contracept Reprod Med. 2022 Sep 14;7:19. doi: 10.1186/s40834-022-00185-w (PMC9471037; doi:10.1186/s40834-022-00185-w)
Supplement: Supplementary file 5 — Additional file 5: Table. Profile of youth respondents of age 15-24. [file 40834_2022_185_MOESM5_ESM.docx]

**Table: Profile of youth respondents of age 15-24**

| **Variable** | **Number** | \| **Percentage** \| \| --- \| |
| --- | --- | --- | --- |
| **Socio-demographic variables** | | |
| **Age**  15-19 years  20-24 years | 515  542 | 48.7  51.3 |
| **Gender**  Male  Female | 472  585 | 44.7  55.3 |
| **Living arrangement of respondent**  With parents  Away from parents | 964  93 | 91.2  8.8 |
| **Sector**  Urban  Estate | 407  345 | 38.5  32.6 |
| Rural | 305 | 28.9 |
| **Ethnicity**  Sinhala  Sri Lanka Tamil  Indian Tamil  Moor  Other | 621  189  108  136  3 | 58.8  17.9  10.2  12.8  0.3 |
| **Religion**  Buddhist  Hindu  Muslim  Christian  Other | 524  213  136  181  3 | 49.6  20.2  12.8  17.1  0.3 |
| **Respondent’s main activity**  Full time student (School)  Full time student (Technical/University)/ etc.  Employed (Govt./Private)  Self-employed (Three-wheel taxi driver)  Self-employed (Other)  Searching employment  No engagement | 150  511  58  21  46  198  73 | 14.2  48.3  5.5  2.0  4.4  18.7  6.9 |
| **Highest education attained**  Primary (1 -5 years)  Secondary (6 – 10 years)  Passed G. C. E. (O. L)  Passed G. C. E. (A .L)  Professional/technical  Undergraduate  Diploma/Degree | 6  48  457  321  114  81  30 | 0.6  4.5  43.2  30.4  10.8  7.7  2.8 |
