# Supplementary material for: Unmarried Sri Lankan youth: sexual behaviour and contraceptive use
Source: Contracept Reprod Med. 2022 Sep 14;7:19. doi: 10.1186/s40834-022-00185-w (PMC9471037; doi:10.1186/s40834-022-00185-w)
Supplement: Supplementary file 6 — Additional file 6: Table. Sexual behavior and contraceptive use by sex. [file 40834_2022_185_MOESM6_ESM.docx]

**Table:** Sexual behavior and contraceptive use by sex

| Variable | Sex | | | | | | | |
| --- | --- | --- | --- | --- | --- | --- | --- | --- |
|  | Female | | | Male | | | Total | |
|  | No | % | | No | % | | No | % |
| **Experience of masturbation** | | | | | | | | |
| Yes | 128 | 29.2 | | 311 | 70.8 | | 439 | 100.0 |
| No | 457 | 73.9 | | 161 | 26.1 | | 618 | 100.0 |
| **Awareness of sexual intercourse by Peers** | | | | | | | | |
| Yes | 194 | 43.5 | | 252 | 56.5 | | 446 | 100.0 |
| No | 237 | 68.3 | | 110 | 31.7 | | 347 | 100.0 |
| Don't know | 154 | 58.3 | | 110 | 41.7 | | 264 | 100.0 |
| **Sexual intercourse experience of respondents** | | | | | | | | |
| Yes | 66 | 35.9 | | 118 | 64.1 | | 184 | 100.0 |
| No | 519 | 59.5 | | 354 | 40.5 | | 873 | 100.0 |
| Total | 585 | 55.3 | | 472 | 44.7 | | 1,057 | 100.0 |
| **Use of Contraception*** | | | | | | | | |
| Never used | 24 | 43.6 | 31 | | | 56.4 | 55 | 100.0 |
| Used | 42 | 32.6 | 87 | | | 67.4 | 129 | 100.0 |
| Total | 66 | 35.9 | 118 | | | 64.1 | 184 | 100.0 |

* Out of respondents with the experience of sexual intercourse (N=184)
