# Supplementary material for: Unmarried Sri Lankan youth: sexual behaviour and contraceptive use
Source: Contracept Reprod Med. 2022 Sep 14;7:19. doi: 10.1186/s40834-022-00185-w (PMC9471037; doi:10.1186/s40834-022-00185-w)
Supplement: Supplementary file 7 — Additional file 7: Table. Type of partner involved in sexual intercourse among unmarried youth. [file 40834_2022_185_MOESM7_ESM.docx]

**Table: Type of partner involved in sexual intercourse among unmarried youth***

| **Sex partner** | **Age in group** | | | | | |
| --- | --- | --- | --- | --- | --- | --- |
|  | **15-19 years** | | **20-24 years** | | **15-24 years** | |
|  | **Male** | **Female** | **Male** | **Female** | **Male** | **Female** |
|  | **N (%)** | **N (%)** | **N (%)** | **N (%)** | **N (%)** | **N (%)** |
| Lover | 46(73.0) | 18(85.7) | 50(90.9) | 39(86.7) | 96(81.4) | 57(86.4) |
| Other friend | 8(12.7) | 3(14.3) | 7(12.7) | 1(2.2) | 15(12.7) | 4(6.1) |
| Commercial sex worker | 4(6.3) | 0(0.0) | 8(14.5) | 1(2.2) | 12(10.2) | 1(1.5) |
| Relative | 3(4.8) | 1(4.8) | 3(5.5) | 1(2.2) | 6(5.1) | 2(3.0) |
| Unknown person | 6(9.5) | 0(0.0) | 0(0.0) | 0(0.0) | 6(5.1) | 0(0.0) |
| Other | 4(6.3) | 3(14.3) | 0(0.0) | 6(13.3) | 4(3.4) | 9(13.6) |
| N | 63 | 21 | 55 | 45 | 118 | 66 |

**Note: Not adding up to 100% due to multiple answers -* some youth had more than one sex partner.

*Source: Survey data.*
