# Supplementary material for: Unmarried Sri Lankan youth: sexual behaviour and contraceptive use
Source: Contracept Reprod Med. 2022 Sep 14;7:19. doi: 10.1186/s40834-022-00185-w (PMC9471037; doi:10.1186/s40834-022-00185-w)
Supplement: Supplementary file 8 — Additional file 8: Table. Use of contraception and selected variables among unmarried youth among unmarried youth who ever had sexual intercourse (N = 184). [file 40834_2022_185_MOESM8_ESM.docx]

**Table: Use of contraception and selected variables among unmarried youth** **among unmarried youth who ever had sexual intercourse (N=184)**

| Variable | Level | Contraceptive use | | | | | |
| --- | --- | --- | --- | --- | --- | --- | --- |
|  |  | Never used | | Used | | Total | |
|  |  | No | % | No | % | No | % |
| Age in group | 20-24 years | 21 | 21.0 | 79 | 79.0 | 100 | 100.0 |
|  | 15-19 years | 34 | 40.5 | 50 | 59.5 | 84 | 100.0 |
| Sex | Female | 24 | 36.4 | 42 | 63.6 | 66 | 100.0 |
|  | Male | 31 | 26.3 | 87 | 73.7 | 118 | 100.0 |
| Sector | Urban | 25 | 30.5 | 57 | 69.5 | 82 | 100.0 |
|  | Rural | 7 | 12.7 | 48 | 87.3 | 55 | 100.0 |
|  | Estate | 23 | 48.9 | 24 | 51.1 | 47 | 100.0 |
| Level of education | Grade 1-10 | 3 | 33.3 | 6 | 66.7 | 9 | 100.0 |
|  | Passed GCE(O/L) | 26 | 31.3 | 57 | 68.7 | 83 | 100.0 |
|  | Passed GCE(A/L) | 11 | 23.4 | 36 | 76.6 | 47 | 100.0 |
|  | Technical/Dip/Undergraduate/Degree | 15 | 33.3 | 30 | 66.7 | 45 | 100.0 |
| Ever had love affair | Yes | 46 | 26.4 | 128 | 73.6 | 174 | 100.0 |
|  | No | 9 | 90.0 | 1 | 10.0 | 10 | 100.0 |
|  | Total | 55 | 29.9 | 129 | 70.1 | 184 | 100.0 |
