# Supplementary material for: Unmarried Sri Lankan youth: sexual behaviour and contraceptive use
Source: Contracept Reprod Med. 2022 Sep 14;7:19. doi: 10.1186/s40834-022-00185-w (PMC9471037; doi:10.1186/s40834-022-00185-w)
Supplement: Supplementary file 9 — Additional file 9: Table. Method of contraception used by age and sex among unmarried youth who ever had sexual intercourse. [file 40834_2022_185_MOESM9_ESM.docx]

**Table: Method of contraception used by age and sex** **among unmarried youth who ever had sexual intercourse**

| **Method(s)** | **Age in group** | | | | | |
| --- | --- | --- | --- | --- | --- | --- |
|  | **15-19 years** | | **20-24 years** | | **15-24 years** | |
|  | **Male** | **Female** | **Male** | **Female** | **Male** | **Female** |
|  | **%** | **%** | **%** | **%** | **%** | **%** |
| Pills | 4.8 | 23.8 | 7.3 | 28.9 | 5.9 | 27.3 |
| Condom | 50.8 | 23.8 | 69.1 | 31.1 | 59.3 | 28.8 |
| Prostinor | 3.2 | 14.3 | 10.9 | 22.2 | 6.8 | 19.7 |
| Natural methods | 9.5 | 23.8 | 34.5 | 26.7 | 21.2 | 25.8 |
| Other methods | 1.6 | .0 | 12.7 | 15.6 | 6.8 | 10.6 |
| N | 63 | 21 | 55 | 45 | 118 | 66 |

*Note: Not adding up to 100% due to multiple answers.*

*Source: Survey data.*
